# Causal relationships between COVID-19 and osteoporosis: a two-sample Mendelian randomization study in European population

**DOI:** 10.3389/fpubh.2023.1122095

**Published:** 2023-05-24

**Authors:** Kai Zhang, Wei Shi, Xinglong Zhang, Ran Pang, Xinyu Liang, Qian Xu, Chunlei Xu, Xin Wan, Wenhao Cui, Dong Li, Zhaohui Jiang, Zhengxuan Liu, Hui Li, Huafeng Zhang, Zhijun Li

**Affiliations:** ^1^Department of Orthopedics, Tianjin Medical University General Hospital, Tianjin, China; ^2^Department of Orthopedics, Sanmenxia Yellow River Hospital, Sanmenxia, China; ^3^School of Integrative Medicine, Tianjin University of Traditional Chinese Medicine, Tianjin, China; ^4^Department of Pharmacology, Kyoto Prefectural University of Medicine, Kyoto, Japan; ^5^R&D Center, Youjia (Hangzhou) Biomedical Technology Co., Ltd., Hangzhou, China; ^6^Department of Orthopedics, Tianjin Nankai Hospital, Tianjin, China

**Keywords:** coronavirus disease 2019, causal effect, genetic, Mendelian randomization, osteoporosis

## Abstract

**Introduction:**

The causal relationship between Coronavirus disease 2019 (COVID-19) and osteoporosis (OP) remains uncertain. We aimed to assess the effect of COVID-19 severity (severe acute respiratory syndrome coronavirus 2 (SARS-CoV-2) infection, COVID-19 hospitalization, and severe COVID-19) on OP by a two-sample Mendelian randomization (MR) study.

**Methods:**

We conducted a two-sample MR analysis using publicly available genome-wide association study (GWAS) data. Inverse variance weighting (IVW) was used as the main analysis method. Four complementary methods were used for our MR analysis, which included the MR–Egger regression method, the weighted median method, the simple mode method, and the weighted mode method. We utilized the MR-Egger intercept test and MR pleiotropy residual sum and outlier (MR-PRESSO) global test to identify the presence of horizontal pleiotropy. Cochran’s Q statistics were employed to assess the existence of instrument heterogeneity. We conducted a sensitivity analysis using the leave-one-out method.

**Results:**

The primary results of IVW showed that COVID-19 severity was not statistically related to OP (SARS-CoV-2 infection: OR (95% CI) = 0.998 (0.995 ~ 1.001), *p* = 0.201403; COVID-19 hospitalization: OR (95% CI) =1.001 (0.999 ~ 1.003), *p* = 0.504735; severe COVID-19: OR (95% CI) = 1.000 (0.998 ~ 1.001), *p* = 0.965383). In addition, the MR-Egger regression, weighted median, simple mode and weighted mode methods showed consistent results. The results were robust under all sensitivity analyses.

**Conclusion:**

The results of the MR analysis provide preliminary evidence that a genetic causal link between the severity of COVID-19 and OP may be absent.

## 1. Introduction

The coronavirus disease 2019 (COVID-19) is a global pandemic caused by the severe acute respiratory syndrome coronavirus 2 (SARS-CoV-2), which has rapidly spread across the world (1). To date, the global count of confirmed COVID-19 cases has surpassed 676.5 million, with over 6.88 million deaths reported (source: https://coronavirus.jhu.edu/map.html). Patients who have recovered from COVID-19 are gradually reporting various complications, such as fatigue, a loss of the sense of smell or taste, impaired pulmonary function, neurological diseases and bone loss (2, 3). Therefore, considering the high infectivity of SARS-CoV-2 and severe adverse consequences of COVID-19, it is necessary to understand the causal relationship between COVID-19 and complications and to take effective preventive measures.

Recent study findings suggest that there is a close correlation between COVID-19 and osteoporosis (OP) (4). OP is a metabolic bone disease that is characterized by a reduction in bone mineral density (BMD), which can lead to bone fragility and an increased risk of fractures (5, 6). However, current research related to COVID-19 and OP is mainly focused on how to prevent and treat OP in patients with COVID-19 (7, 8), and there is a lack of large prospective cohort studies or randomized controlled trials (RCTs) to evaluate the effect of COVID-19 on OP. In addition, RCTs for COVID-19 are difficult to conduct due to the need for extensive human resources and time-consuming follow-up. Furthermore, existing observational studies may have biased conclusions due to the possibility of confounders. To minimize the impact of confounders on the association between COVID-19 and OP, a more efficient method for inferring potential causal relationships is needed.

**Figure 1:**
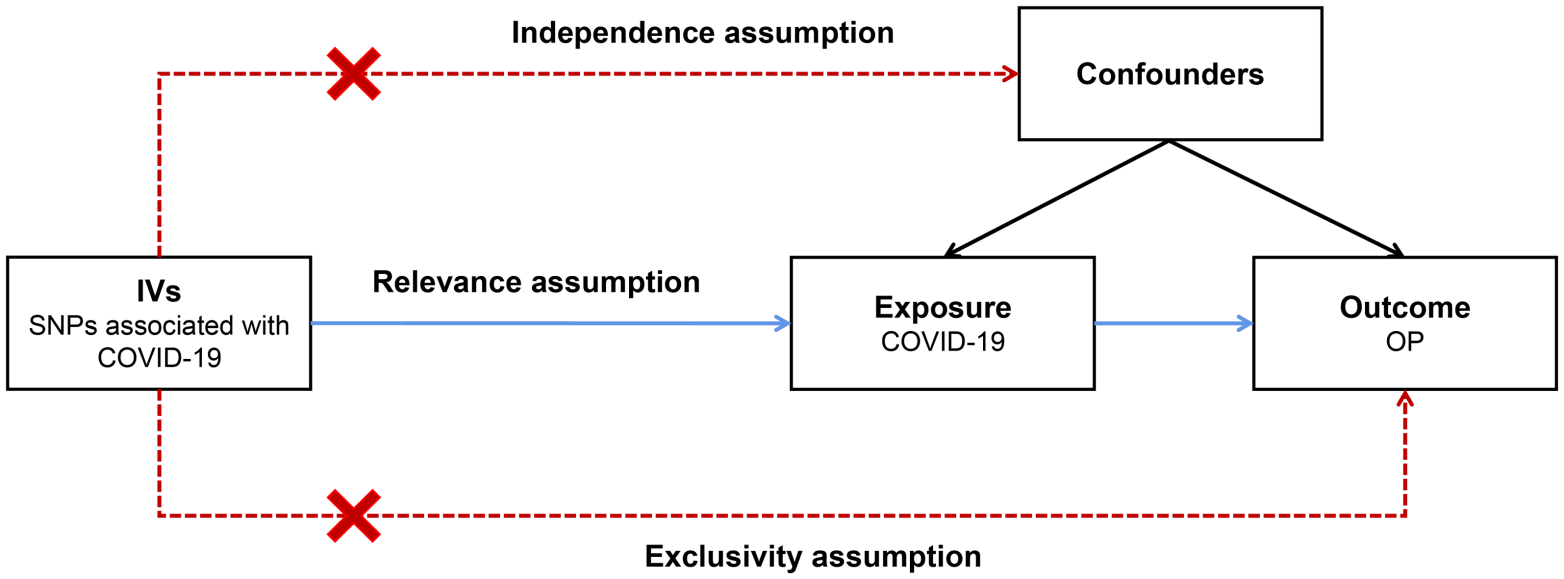
Schematic of a two-sample Mendelian randomization (MR) study. The IVs in an MR analysis should satisfy the three core assumptions of relevance, independence and exclusivity; that is, (1) the IVs should be strongly correlated with exposure, (2) the IVs should independent of confounders that affect the exposure-outcome relationship, and (3) the IVs should be capable of affecting the outcome only through exposure and have no direct correlation with the outcome. IVs: instrumental variables; SNPs: single nucleotide polymorphisms; COVID-19: coronavirus disease 2019; OP: osteoporosis.

In recent years, Mendelian randomization (MR) analysis has been widely used in causal inference in epidemiology (9, 10). The main principle behind this approach involves utilizing genetic variants as instrumental variables (IVs) to establish a causal relationship between exposure and outcome (11). As shown in Figure 1, the IVs in an MR analysis should satisfy the three core assumptions of relevance, independence and exclusivity (12); that is, (1) the IVs should be strongly correlated with exposure, (2) the IVs should be independent of confounders that affect the exposure-outcome relationship, and (3) the IVs should be capable of affecting the outcome only through exposure and have no direct correlation with the outcome. Ran et al. conducted a two-sample MR analysis for the association of total body BMD with severe COVID-19 (13). Their results indicate that BMD might be a useful predictor of severe COVID-19 in older adult populations and could help identify individuals at higher risk of disease progression. However, it did not delve into the impact of COVID-19 on osteoporosis.

As there is currently no conclusive proof to establish a causal relationship between COVID-19 and OP, we used single nucleotide polymorphisms (SNPs) strongly associated with COVID-19 severity (SARS-CoV-2 infection, COVID-19 hospitalization, and severe COVID-19) as IVs, and we carried out a two-sample MR analysis using genome-wide association study (GWAS) summary statistics to explore the causal effect of COVID-19 severity on OP.

## 2. Methods

### 2.1. Definitions

SARS-CoV-2 infection was defined as laboratory-confirmed infection with SARS-CoV-2 with or without symptoms. COVID-19 hospitalization was defined as first hospital admission between 7 days before and 15 days after the first COVID-19 positive date. Severe COVID-19 was defined as dyspnea, respiratory rate ≤ 30/min, SpO_2_ ≤ 93%, PaO_2_/FiO_2_ < 300 mmHg, or more than 50% infiltration of the lung fields (14). Osteoporosis was defined as a BMD T-score ≤ −2.5 at any anatomical site (15).

### 2.2. Study design

**Figure 2:**
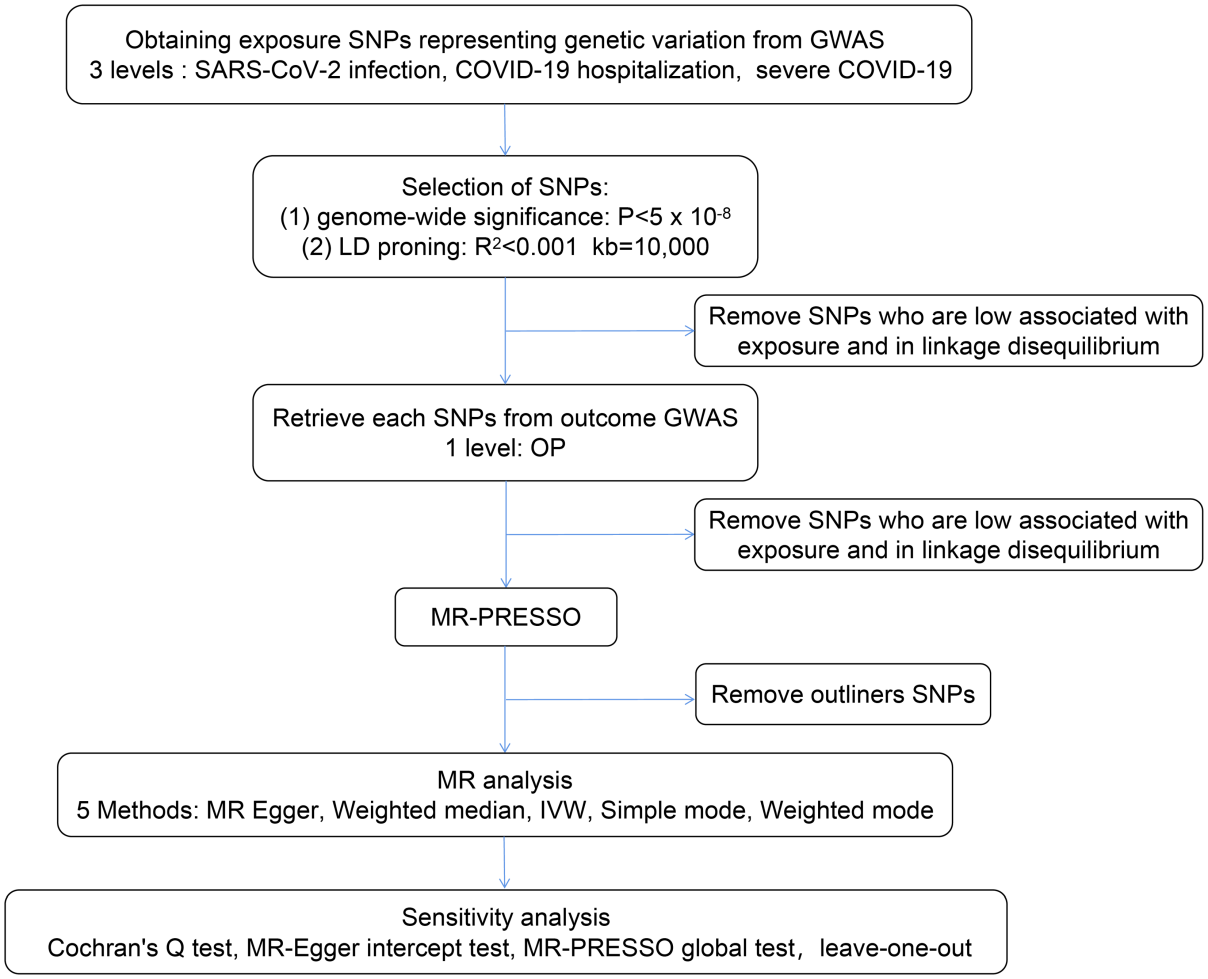
Flow chart of the MR study design. A two-sample MR analysis was performed to examine the causal effects of COVID-19 severity (severe acute respiratory syndrome coronavirus 2 (SARS-CoV-2) infection, COVID-19 hospitalization, and severe COVID-19) on OP using GWAS summary statistics, and the reliability of the results was tested. LD, linkage disequilibrium; IVW, inverse variance weighted; MR-PRESSO, Mendelian randomization pleiotropy residual sum and outlier; GWAS, genome wide association study.

In this study, we performed a two-sample MR analysis to examine the causal effects of COVID-19 severity (SARS-CoV-2 infection, COVID-19 hospitalization, and severe COVID-19) on OP using GWAS summary statistics, and we tested the reliability of the results (Figure 2).

### 2.3. Data source

In this two-sample MR study, the exposures were SARS-CoV-2 infection (cases:controls = 38,984:1,644,784), COVID-19 hospitalization (cases:controls = 9,986:1,877,672) and severe COVID-19 (cases:control = 5,101:1,383,241), and the outcome was OP (cases:controls = 7,547: 455,386). GWAS summary statistics of exposure were obtained from the COVID-19 Host Genetic Initiative (HGI) (Round 5) (16). Summary statistics of OP were extracted from a GWAS conducted in UK Biobank^1^ participants. Detailed information about the aggregated GWAS results is shown in Table 1.

**Table 1:** Detailed information about the aggregated GWAS results.

GWAS ID	Trait	Sample size	SNPs (*n*)	Cases (*n*)	Controls (*n*)	Population
ebi-a-GCST011073	SARS-CoV-2 infection	1,683,768	8,660,177	38,984	1,644,784	European
ebi-a-GCST011081	COVID-19 hospitalization	1,887,658	8,107,040	9,986	1,877,672	European
ebi-a-GCST011075	Severe COVID-19	1,388,342	9,739,225	5,101	1,383,241	European
ukb-b-12141	Osteoporosis	462,933	9,851,867	7,547	455,386	European

GWAS, genome-wide association study; SNPs, single nucleotide polymorphisms; SARS-CoV-2, severe acute respiratory syndrome coronavirus 2; COVID-19, coronavirus disease 2019.

### 2.4. Selection of the genetic instruments

To filter eligible genetic IVs that fulfilled the three core MR assumptions, we performed a set of quality control techniques. Independent SNPs associated with genome-wide exposure (*p* < 5 × 10^−8^) were selected as instrumental SNPs according to the three assumptions of MR analysis, and parameters (R^2^ < 0.001 and kb = 10,000) were set to exclude SNPs with strong linkage disequilibrium (LD) (17). To verify that the selected IVs satisfied the independence assumptions, a study was conducted by PhenoScanner^2^ to check whether the remaining SNPs were associated with other phenotypes. An MR pleiotropy residual sum and outlier (MR-PRESSO) test was performed to detect and remove outlier instruments (18).

### 2.5. Statistical analysis

#### 2.5.1. Weak IV test

The hypothesis of association was further tested by calculating the F-statistic to assess the presence of weak IV bias in the selected IVs (19).

#### 2.5.2. MR analyses and power calculations

The main statistical approach used to evaluate the relationship between COVID-19 severity and OP was the inverse variance weighted (IVW) method. This method is commonly used in MR studies and is known for providing robust causal estimates, even in the absence of directional pleiotropy (20). A *p* value below 2.78E−03 (0.05/18) after Bonferroni correction was considered statistically significant. In addition, other methods were used to complement the MR results, including the MR–Egger regression method, the weighted median method, the simple mode method and the weighted mode method. The power calculations were carried out utilizing the online tool available at https://shiny.cnsgenomics.com/mRnd/ (21).

#### 2.5.3. Evaluation of reliability

The MR-Egger intercept test and MR-PRESSO global test were used to detect the presence of horizontal pleiotropy (22). Cochran’s Q statistics were used to reflect the presence of the heterogeneity of instruments. A sensitivity analysis of the results was performed separately using the leave-one-out method.

#### 2.5.4. Software and pre-registration

All analyses were carried out using the “TwoSampleMR” (23) and “MRPRESSO” packages in R version 4.0.3. Since the study was based on existing publications and public databases, and therefore did not require additional ethical approval or consent.

## 3. Results

### 3.1. Results of SNPs and the weak IV test

Finally, 7 SNPs for SARS-CoV-2 infection-OP, 5 SNPs for COVID-19 hospitalization-OP, and 7 SNPs (A missing SNP was deleted) for severe COVID-19-OP were used as the IVs, and the *F* values were 4,560.3626, 28,378.09481 and 40,410.013, respectively (Tables 2–4). Weak IVs are less likely to occur when *F* > 10 (19), so the results of the MR analysis were not likely to be affected by weak IV bias.

**Table 2:** Summary genetic instruments between SARS-CoV-2 infection and OP.

SNP	Chr	EA	OA	SARS-CoV-2 infection				OP				*R* ^2^	*F*
				eaf	beta	se	pval	eaf	beta	se	pval		
rs10936744	3	T	C	0.3588	−0.062641	0.0099836	3.51E-10	0.355314	4.61E-05	0.000274	0.87	0.001805483	39.36796987
rs12482060	21	G	C	0.3375	0.061951	0.010525	3.96E-09	0.310041	3.98E-05	0.000284	0.89	0.001716273	34.64594671
rs17078348	3	G	A	0.0997	0.092084	0.016154	1.20E-08	0.089067	0.000807	0.000467	0.084	0.001522232	32.49437553
rs2271616	3	T	G	0.1181	0.15634	0.015084	3.61E-25	0.133113	−0.00022	0.00039	0.57	0.005091426	107.4254466
rs4971066	1	G	T	0.1777	−0.076762	0.0134	1.02E-08	0.162048	0.000345	0.000357	0.33	0.001722028	32.81579775
rs643434	9	A	G	0.371	0.1013	0.010114	1.29E-23	0.341373	−0.00038	0.000276	0.16	0.004789315	100.3166434
rs757405	12	A	T	0.7092	0.068926	0.010783	1.64E-10	0.673961	−0.00046	0.00028	0.097	0.001959563	40.85892546
												=0.01860632	=4560.3626

Chr, chromosome; EA, effector allele; OA, noneffector allele; eaf, effector allele frequency; pval, *p* value; beta, allele effect value; se, standard error; SARS-CoV-2, severe acute respiratory syndrome coronavirus 2; OP, osteoporosis.

**Table 3:** Summary genetic instruments between COVID-19 hospitalization and OP.

SNP	Chr	EA	OA	COVID-19 hospitalization				OP				*R* ^2^	*F*
				eaf	beta	se	pval	eaf	beta	se	pval		
rs505922	9	C	T	0.3501	0.11182	0.019056	4.42E-09	0.317868	−0.00028	0.000281	0.32	0.005689939	34.43304955
rs35081325	3	T	A	0.08122	0.48825	0.031508	3.68E-54	0.069989	0.001185	0.000515	0.021	0.035578613	240.1280147
rs2660	12	A	G	0.6902	0.11639	0.019406	2.00E-09	0.646381	−0.00032	0.000274	0.25	0.005793189	35.97156081
rs2109069	19	A	G	0.3227	0.15131	0.019906	2.94E-14	0.322627	−8.92E-05	0.000281	0.75	0.010007954	57.77863307
rs13050728	21	C	T	0.6528	−0.16832	0.020183	7.44E-17	0.690011	−4.12E-05	0.000284	0.88	0.012842847	69.55045965
												=0.06991254	=28378.09481

Chr, chromosome; EA, effector allele; OA, noneffector allele; eaf, effector allele frequency; beta, allele effect value; se, standard error; COVID-19, coronavirus disease 2019; OP, osteoporosis.

**Table 4:** Summary genetic instruments between severe COVID-19 and OP.

SNP	Chr	EA	OA	Severe COVID-19				OP				*R* ^2^	*F*
				eaf	beta	se	pval	eaf	beta	se	pval		
rs10860891	12	A	C	0.8855	−0.23948	0.039713	1.64E-09	0.893414	−0.00023	0.000427	0.59	0.01162954	36.3641221
rs111837807	6	C	T	0.0996	0.29453	0.04276	5.66E-12	0.065527	−0.00098	0.000529	0.065	0.015559079	47.44426049
rs13050728	21	C	T	0.6627	−0.20011	0.028559	2.44E-12	0.690011	−4.12E-05	0.000284	0.88	0.017901973	49.09661959
rs2109069	19	A	G	0.3287	0.25663	0.02807	6.12E-20	0.322627	−8.92E-05	0.000281	0.75	0.029064389	83.58532254
rs2237698	7	T	C	0.08971	0.23662	0.039653	2.41E-09	0.081985	−0.00047	0.000464	0.31	0.009144364	35.60826391
rs2384074	12	T	C	0.6756	0.19824	0.02821	2.10E-12	0.647022	−0.00058	0.000275	0.036	0.017225945	49.38287903
rs35081325	3	T	A	0.07529	0.62617	0.044502	5.75E-45	0.069989	0.001185	0.000515	0.021	0.054595564	197.9818848
rs77534576	17	T	C	0.03465	0.45975	0.074941	8.52E-10					0.014140394	37.63609083
												=0.169261249	=40410.013

Chr, chromosome; EA, effector allele; OA, noneffector allele; eaf, effector allele frequency; beta, allele effect value; se, standard error; COVID-19, coronavirus disease 2019; OP, osteoporosis.

### 3.2. Results of the two-sample MR analyses

**Figure 3:**
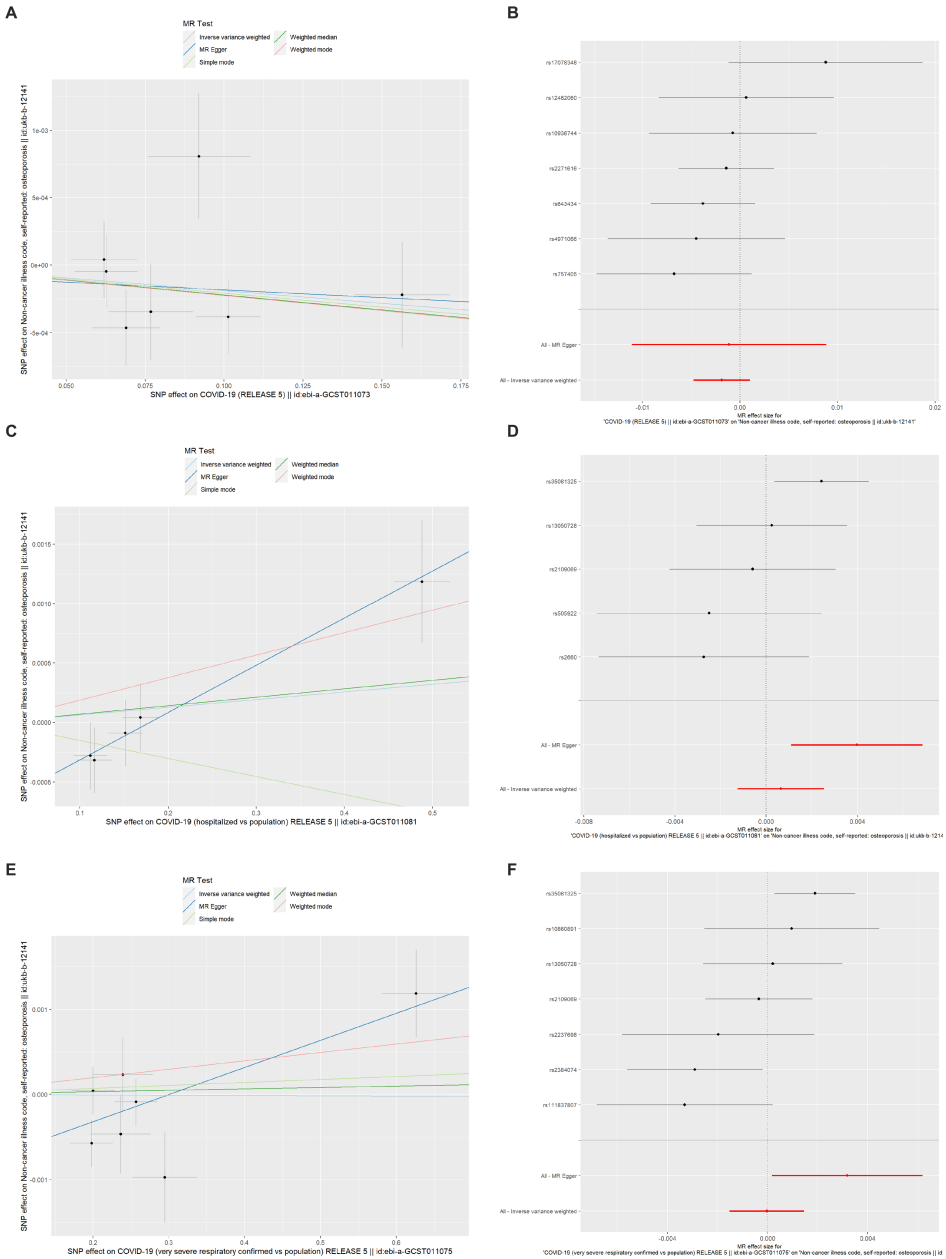
Scatter plot and forest plot of the causal relationships between COVID-19 severity and OP using different MR methods. **(A)** Scatter plot of the causal relationships between SARS-CoV-2 infection and OP; **(B)** Forest plot of the causal relationships between SARS-CoV-2 infection and OP; **(C)** Scatter plot of the causal relationships between COVID-19 hospitalization and OP; **(D)** Forest plot of the causal relationships between COVID-19 hospitalization and OP; **(E)** Scatter plot of the causal relationships between severe COVID-19 and OP; **(F)** Forest plot of the causal relationships between severe COVID-19 and OP. The slope of each line corresponds to the causal estimates for each method. The individual SNP effect on the outcome (point and vertical line) against its effect on the exposure (point and horizontal line) was delineated in the background. SARS-CoV-2, severe acute respiratory syndrome coronavirus 2; COVID-19, coronavirus disease 2019; MR, Mendelian randomization; OP, osteoporosis; SNP, single nucleotide polymorphism.

The MR estimates of different methods are presented in Table 5 and Figure 3. Overall, there were no causal associations between COVID-19 severity (SARS-CoV-2 infection, COVID-19 hospitalization, and severe COVID-19) and OP. The primary results of the IVW analysis showed that COVID-19 severity was not statistically related to OP (SARS-CoV-2 infection: OR (95% CI) = 0.998 (0.995 ~ 1.001), *p* = 0.201403; COVID-19 hospitalization: OR (95% CI) =1.001 (0.999 ~ 1.003), *p* = 0.504735; severe COVID-19: OR (95% CI) = 1.000 (0.998 ~ 1.001), *p* = 0.965383). In addition, the MR–Egger regression, weighted median, simple mode and weighted mode methods showed consistent results.

**Table 5:** Results of the two-sample MR analyses.

Exposure	Outcome	Method	SNPs (*n*)	beta	se	pval	OR (95% CI)	MR power
SARS-CoV-2 infection	Osteoporosis	IVW	7	−0.001884	0.0014745	0.201403	0.998 (0.995 ~ 1.001)	0.05
		MR Egger	7	−0.001145	0.0050997	0.8312342	0.999 (0.989 ~ 1.009)	0.05
		Weighted median	7	−0.002209	0.0017027	0.1945684	0.998 (0.994 ~ 1.001)	0.05
		Simple mode	7	−0.002075	0.0023558	0.412263	0.998 (0.993 ~ 1.003)	0.05
		Weighted mode	7	−0.002242	0.001972	0.2988885	0.998 (0.994 ~ 1.002)	0.05
COVID-19 hospitalization	Osteoporosis	IVW	5	0.000642	0.000963	0.504735	1.001 (0.999 ~ 1.003)	0.05
		MR Egger	5	0.003973	0.00147	0.073618	1.004 (1.001 ~ 1.007)	0.05
		Weighted median	5	0.000709	0.000988	0.473038	1.001 (0.999 ~ 1.003)	0.05
		Simple mode	5	−0.00151	0.001799	0.447895	0.998 (0.995 ~ 1.002)	0.05
		Weighted mode	5	0.001889	0.001001	0.132165	1.002 (1.000 ~ 1.004)	0.05
Severe COVID-19	Osteoporosis	IVW	7	−3.29E-05	0.000757	0.965383	1.000 (0.998 ~ 1.001)	0.05
		MR Egger	7	0.003184	0.001533	0.092444	1.003 (1.000 ~ 1.006)	0.05
		Weighted median	7	0.000164	0.000749	0.827061	1.000 (0.999 ~ 1.002)	0.05
		Simple mode	7	0.000351	0.001487	0.821238	1.000 (0.997 ~ 1.003)	0.05
		Weighted mode	7	0.000986	0.000946	0.337556	1.001 (0.999 ~ 1.003)	0.05

MR, Mendelian randomization; SNPs, single nucleotide polymorphisms; pval, *p* value; beta, allele effect value; se, standard error; SARS-CoV-2, severe acute respiratory syndrome coronavirus 2; COVID-19, coronavirus disease 2019; IVW, inverse variance weighted.

### 3.3. Evaluation of reliability

**Figure 4:**
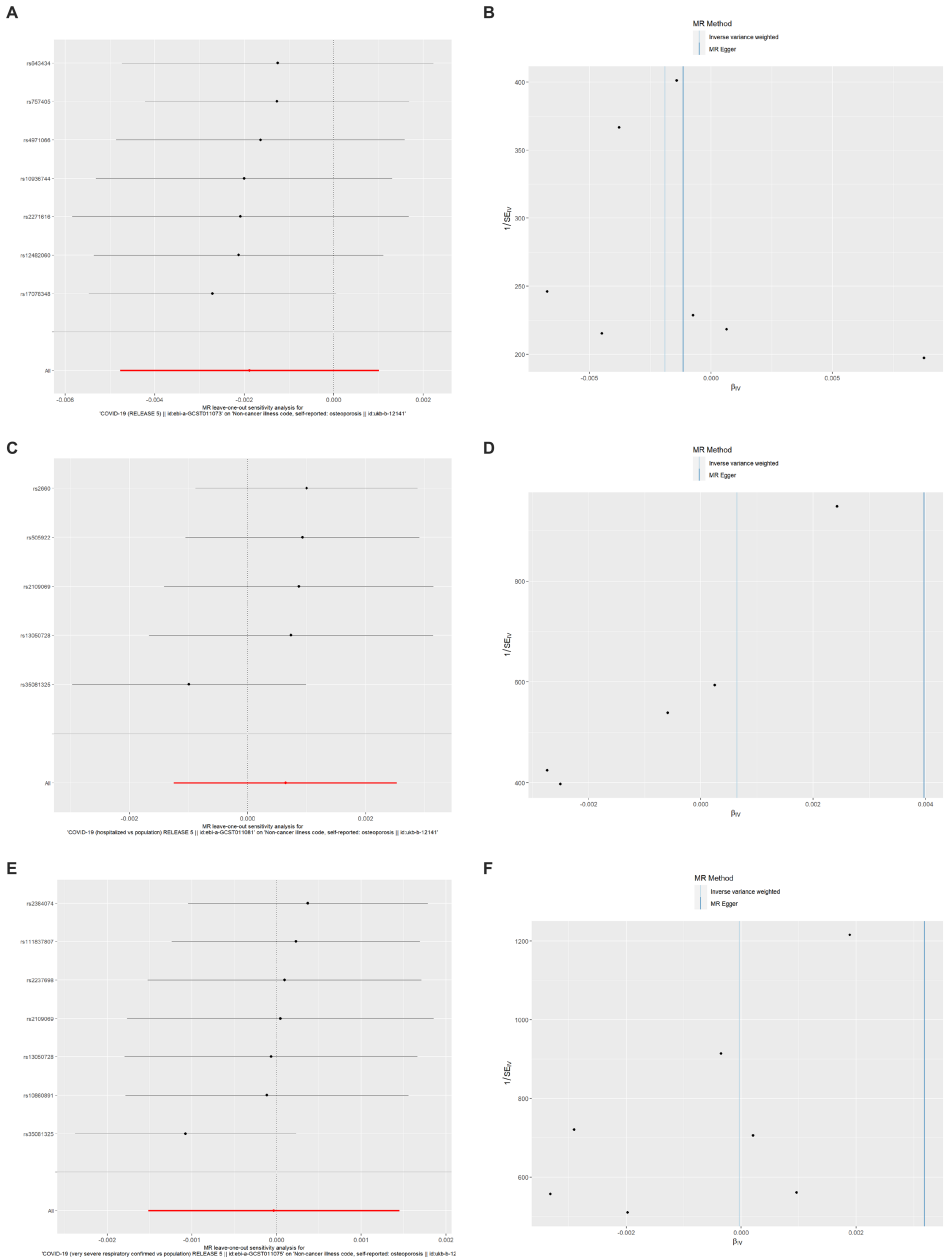
Results of leave-one-out method sensitivity analysis and funnel plots. **(A)** Leave-one-out sensitivity analysis for the effect of SARS-CoV-2 infection on OP; **(B)** Funnel plot for the effect of SARS-CoV-2 infection on OP analysis; **(C)** Leave-one-out sensitivity analysis for the effect of COVID-19 hospitalization on OP; **(D)** Funnel plot for the effect of COVID-19 hospitalization on OP analysis; **(E)** Leave-one-out sensitivity analysis for the effect of severe COVID-19 on OP; **(F)** Funnel plot for the effect of severe COVID-19 on OP analysis. SARS-CoV-2, severe acute respiratory syndrome coronavirus 2; COVID-19, coronavirus disease 2019; OP, osteoporosis.

As shown in Table 6, the results of the MR-Egger intercept, Cochran’s Q heterogeneity, and MR-PRESSO global tests were all statistically nonsignificant, indicating that the MR analysis results were reliable. The results of the leave-one-out method showed that after gradually removing each SNP, the results with remaining SNPs were similar to the original results, with a *p* value>0.05 (Figures 4A,C,E), and the funnel plots appeared generally symmetrical (Figures 4B,D,F), indicating that no SNPs with a strong influence on the results were found in the IVs.

**Table 6:** Reliability test of MR analysis results.

Exposure	Outcome	Method	Cochran’s Q heterogeneity test *p*	MR-Egger intercept test *p*	MR-PRESSO global test *p*
SARS-CoV-2 infection	Osteoporosis	IVW	0.3152750	0.8846124	0.392
		MR Egger	0.2185753		
COVID-19 hospitalization	Osteoporosis	IVW	0.9828618	0.07972223	0.212
		MR Egger	0.1371936		
Severe COVID-19	Osteoporosis	IVW	0.20973942	0.07261179	0.061
		MR Egger	0.02446938		

MR, Mendelian randomization; SARS-CoV-2, severe acute respiratory syndrome coronavirus 2; COVID-19, coronavirus disease 2019; IVW, inverse variance weighted; MR-PRESSO, MR pleiotropy residual sum and outlier.

## 4. Discussion

There is a lack of large prospective cohort studies or RCTs to evaluate the effect of COVID-19 on OP. Furthermore, existing studies may have biased conclusions due to the possibility of confounders. To minimize the impact of confounders on the association between COVID-19 and OP, we used SNPs strongly associated with COVID-19 severity (SARS-CoV-2 infection, COVID-19 hospitalization, and severe COVID-19) as IVs and carried out a two-sample MR analysis using GWAS summary statistics, and we obtained more reliable results. Because genetic variants are formed *in utero* and remain with the person throughout life, exposure differences throughout life exist between genetic subgroups, and because an exposure precedes an outcome, thus being temporally sequential, reverse causality is less likely. Additionally, the examination of each SNP as an IV revealed that it was associated with the exposure factor COVID-19 but not with other phenotypes, and it is unlikely that it would affect the outcome through other genetic pathways. Identifying the causal relationship between COVID-19 and OP is important for the prevention, diagnosis and treatment of OP. To our knowledge, this is the first two-sample MR study to explore the causal effect of COVID-19 severity on OP.

There could be various factors that potentially explain the link between COVID-19 and OP. Current research suggests that SARS-CoV-2 may affect bone homeostasis by directly or indirectly affecting osteoblasts and osteoclasts, leading to bone loss. The effects of SARS-CoV-2 on humans have been found to be attributable to its binding to the angiotensin-converting enzyme 2 receptor (ACE2) (24). SARS-CoV-2 downregulates ACE2 expression and enhances angiotensin II (Ang II) levels upon the infection of target cells (25). Shimizu et al. demonstrated that Ang II significantly induced the expression of receptor activator of NF-kappaB ligand (RANKL) in osteoblasts, leading to the activation of osteoclasts (26). A cytokine storm might be another important cause of abnormal bone metabolism. Upon SARS-CoV-2 infection, innate immune cells such as macrophages and neutrophils are immediately activated, leading to a limitation of infection. This activation triggers downstream activation of the persistent adaptive immune system, resulting in the production of neutralizing antibodies and T-cell responses against the virus. However, when inflammation serves its purpose and is not resolved, it leads to dysregulated hyperinflammation, a cytokine storm, and the suppression of the adaptive immune system, which further escalates tissue damage and organ failure. A cytokine storm is characterized by the uncontrolled production of multiple inflammatory cytokines, including IL-6, IL-7, IL-2, IL-17, TNF-α, as well as monocyte chemoattractant protein (MCP)-1 and macrophage inhibitory protein (MIP)-1α (27, 28). Qiao et al. demonstrated that bone loss is associated with SARS-CoV-2-induced cytokine dysregulation, as circulating proinflammatory cytokines not only upregulate osteoclastic differentiation in bone tissues but also trigger an amplified proinflammatory cascade in skeletal tissues to augment their pro-osteoclastogenesis effect (29). It has been suggested that hypoxia and oxidative stress may play a role in the development of osteoporosis in COVID-19 patients (30). Severe COVID-19-induced hypoxemia can trigger the excessive production of reactive oxygen species (ROS), which disrupts the balance of redox homeostasis (31). This disruption has been found to induce apoptosis of osteoblasts and osteoclasts, and to regulate the expression of RANKL/osteoprotegerin (OPG), leading to the generation of osteoclasts and ultimately bone loss (32). Glucocorticoids are beneficial in the treatment of acute respiratory distress syndrome (ARDS) by reducing inflammation and improving the function of the lungs and extrapulmonary organs and are therefore widely used in patients with COVID-19 in most parts of the world (33). However, it is considered to be a double-edged sword in the treatment of patients with COVID-19. Glucocorticoids affect bone homeostasis by inhibiting osteoblast osteogenesis and promoting osteoclast resorption, leading to bone loss, and have been found to be the cause of medically induced OP, commonly known as glucocorticoid-induced osteoporosis (GIOP) (34). Therefore, there is a link and interaction among COVID-19, glucocorticoids and osteoporosis that deserves the attention of clinicians and researchers. Another possible reason is that with the COVID-19 pandemic, many countries implemented an unprecedented array of measures to mitigate the spread of the virus, including mass social isolation, travel bans, restrictions on public gatherings, and national lockdowns (35). While these social distancing strategies were necessary from a public health perspective, they presented challenges in the management of many chronic diseases. A study found that physical activity decreased significantly during the COVID-19 pandemic (36), which undoubtedly had an impact on the onset or progression of OP.

In contrast, there was no evidence observed in our two-sample MR analysis to support the association of genetically predicted OP with COVID-19 severity in individuals of European descent based on the results. Therefore, it is suggested that COVID-19 patients may not require special preventive or treatment measures for osteoporosis. However, we cannot exclude the possibility that we failed to detect the association due to the limitation of this study. First, this study used a population sample of European origin and lacked data from other ethnic groups, so the extrapolation of the results is limited, and data from other ethnic groups are needed for analysis and comparison to make the results more reliable. Second, this study used summary GWAS data and was unable to assess the nonlinear relationship between exposure and outcome, and because of the lack of individual data, it was not possible to stratify the analysis by sex, age, or the site of OP. In addition, there were too few SNPs as IVs in this study, which might have some influence on the results, especially for R^2^ and MR power. If a larger sample could be obtained and more SNPs could be extracted, the reliability of the results could be further verified. Furthermore, the severity of COVID-19 is likely to be impacted by a myriad of factors, including the healthcare infrastructure, social contact patterns, environmental conditions, and viral strain mutations, among others. It is important to note that these factors can interact with one another, making it challenging to isolate their individual impacts on the severity of COVID-19. Additionally, these factors are complex and challenging to explain through MR Analysis.

## 5. Conclusion

In summary, the results of the MR analysis provide preliminary evidence that a genetic causal link between the severity of COVID-19 and OP may be absent. Therefore, it is suggested that COVID-19 patients may not require special preventive or treatment measures for osteoporosis. However, the contribution of other factors cannot be dismissed. To corroborate the study’s conclusions, additional MR analyses incorporating more extensive GWAS summary data and a larger set of genetic instruments, coupled with sizable prospective cohort studies or RCTs, are indispensable.

## Data availability statement

The datasets presented in this study can be found in online repositories. The names of the repository/repositories and accession number(s) can be found in the article/Supplementary material.

## Author contributions

KZ as the first author performed data analysis and wrote the manuscript. HL, QX, ZJL, WC, DL, and XW contributed suggestions for manuscript revision and revised the manuscript. WS, XZ, RP, XL, ZJ, ZXL, and CX provided advice and suggestions while we met some problems during the data analysis process. HZ and ZJL conceived and initiated this project, provided advice on experimental design, oversaw the implementation of the statistical method, and revised or finalized the manuscript. All authors contributed to the article and approved the submitted version.

## Conflict of interest

WC was employed by R&D Center, Youjia (Hangzhou) Biomedical Technology Co., Ltd.

The remaining authors declare that the research was conducted in the absence of any commercial or financial relationships that could be construed as a potential conflict of interest.

## Publisher’s note

All claims expressed in this article are solely those of the authors and do not necessarily represent those of their affiliated organizations, or those of the publisher, the editors and the reviewers. Any product that may be evaluated in this article, or claim that may be made by its manufacturer, is not guaranteed or endorsed by the publisher.

## Footnotes

^1^ https://www.ukbiobank.ac.uk/

^2^ http://www.phenoscanner.medschl.cam.ac.uk
