# Supplementary material for: Causal relationships between COVID-19 and osteoporosis: a two-sample Mendelian randomization study in European population
Source: Front Public Health. 2023 May 24;11:1122095. doi: 10.3389/fpubh.2023.1122095 (PMC10244501; doi:10.3389/fpubh.2023.1122095)
Supplement: Supplementary file 1 [file Data_Sheet_1.DOCX]

**The code used for analysis in R**

rm(list=ls())

devtools::install_github("MRCIEU/TwoSampleMR")

library(TwoSampleMR)

library(MRPRESSO)

## effect of SARS-CoV-2 infection on OP analysis

ao <- available_outcomes()

exposure_dat <- extract_instruments(c('ebi-a-GCST011073'),p1 = 5e-08)

dim(exposure_dat)

exposure_dat <- clump_data(exposure_dat,clump_r2=0.001,clump_kb=10000,pop = "EUR")

dim(exposure_dat)

outcome_dat <- extract_outcome_data(exposure_dat$SNP, c('ukb-b-12141'), proxies = 1, rsq = 0.8, align_alleles = 1, palindromes = 1, maf_threshold = 0.3)

dat <- harmonise_data(exposure_dat, outcome_dat, action = 2)

mr_results <- mr(dat)

generate_odds_ratios(mr_results)

mr_method_list()

mr(dat, method_list=c("mr_egger_regression", "mr_ivw","mr_two_sample_ml"))

mr(dat,method_list=c('mr_ivw_mre'))

mr_heterogeneity(dat)

mr_pleiotropy_test(dat)

res <-mr(dat)

p1 <-mr_scatter_plot(res, dat)

p1

res_single <- mr_singlesnp(dat)

p2 <- mr_forest_plot(res_single)

p2

res_single <- mr_singlesnp(dat)

p3 <- mr_funnel_plot(res_single)

p3

p4 <- mr_leaveoneout(dat)

mr_leaveoneout_plot(p4)

mr_presso(BetaOutcome="beta.outcome",BetaExposure="beta.exposure",SdOutcome="se.outcome", SdExposure="se.exposure",OUTLIERtest=TRUE,DISTORTIONtest=TRUE, data=dat, NbDistribution= 1000,SignifThreshold=0.05)

## effect of COVID-19 hospitalization on OP

ao <- available_outcomes()

exposure_dat <- extract_instruments(c('ebi-a-GCST011081'),p1 = 5e-08)

dim(exposure_dat)

exposure_dat <- clump_data(exposure_dat,clump_r2=0.001,clump_kb=10000,pop = "EUR")

dim(exposure_dat)

outcome_dat <- extract_outcome_data(exposure_dat$SNP, c('ukb-b-12141'), proxies = 1, rsq = 0.8, align_alleles = 1, palindromes = 1, maf_threshold = 0.3)

dat <- harmonise_data(exposure_dat, outcome_dat, action = 2)

mr_results <- mr(dat)

generate_odds_ratios(mr_results)

mr_method_list()

mr(dat, method_list=c("mr_egger_regression", "mr_ivw","mr_two_sample_ml"))

mr(dat,method_list=c('mr_ivw_mre'))

mr_heterogeneity(dat)

mr_pleiotropy_test(dat)

res <-mr(dat)

p1 <-mr_scatter_plot(res, dat)

p1

res_single <- mr_singlesnp(dat)

p2 <- mr_forest_plot(res_single)

p2

res_single <- mr_singlesnp(dat)

p3 <- mr_funnel_plot(res_single)

p3

p4 <- mr_leaveoneout(dat)

mr_leaveoneout_plot(p4)

mr_presso(BetaOutcome="beta.outcome",BetaExposure="beta.exposure",SdOutcome="se.outcome", SdExposure="se.exposure",OUTLIERtest=TRUE,DISTORTIONtest=TRUE, data=dat, NbDistribution= 1000,SignifThreshold=0.05)

## effect of severe COVID-19 on OP

ao <- available_outcomes()

exposure_dat <- extract_instruments(c('ebi-a-GCST011075'),p1 = 5e-08)

dim(exposure_dat)

exposure_dat <- clump_data(exposure_dat,clump_r2=0.001,clump_kb=10000,pop = "EUR")

dim(exposure_dat)

outcome_dat <- extract_outcome_data(exposure_dat$SNP, c('ukb-b-12141'), proxies = 1, rsq = 0.8, align_alleles = 1, palindromes = 1, maf_threshold = 0.3)

dat <- harmonise_data(exposure_dat, outcome_dat, action = 2)

mr_results <- mr(dat)

generate_odds_ratios(mr_results)

mr_method_list()

mr(dat, method_list=c("mr_egger_regression", "mr_ivw","mr_two_sample_ml"))

mr(dat,method_list=c('mr_ivw_mre'))

mr_heterogeneity(dat)

mr_pleiotropy_test(dat)

res <-mr(dat)

p1 <-mr_scatter_plot(res, dat)

p1

res_single <- mr_singlesnp(dat)

p2 <- mr_forest_plot(res_single)

p2

res_single <- mr_singlesnp(dat)

p3 <- mr_funnel_plot(res_single)

p3

p4 <- mr_leaveoneout(dat)

mr_leaveoneout_plot(p4)

mr_presso(BetaOutcome="beta.outcome",BetaExposure="beta.exposure",SdOutcome="se.outcome", SdExposure="se.exposure",OUTLIERtest=TRUE,DISTORTIONtest=TRUE, data=dat, NbDistribution= 1000,SignifThreshold=0.05)
